# Editorial: Methods in brain-computer interfaces: 2023

**DOI:** 10.3389/fnhum.2025.1647584

**Published:** 2025-07-09

**Authors:** Davide Borra, Ming Ma, Ester Martinez-Martin, Likun Xia

**Affiliations:** ^1^Department of Electrical, Electronic and Information Engineering “Guglielmo Marconi” (DEI), University of Bologna, Cesena, Italy; ^2^Department of Graduate Computer Science and Engineering, Katz School of Science and Health, Yeshiva University, New York City, NY, United States; ^3^Department of Computer Science and Artificial Intelligence, University of Alicante, Alicante, Spain; ^4^College of Information Engineering, Capital Normal University, Beijing, China

**Keywords:** brain-computer interfaces, assistive technologies, rehabilitation, neural decoding, artificial intelligence

## Introduction

Brain-computer interfaces (BCIs) represent a rapidly evolving field within human neuroscience, enabling direct link between the brain and external devices. BCI research is aiming at transitioning from experimental constructs to accurate and generalized tools with transformative implications in clinical, commercial, and assistive domains. Despite significant advancements in the field, such as the design and application of artificial intelligence methods (e.g., convolutional neural networks, Roy et al., 2019) for improving neural decoding, several challenges persist. A primary concern involves neural decoding, which is affected by intra- and inter-subject variability as well as the limited availability of labeled data (Saha and Baumert, 2020). In addition, improving user experience and ensuring system robustness in complex, real-world scenarios are critical challenges that should be addressed to advance practical BCI applications (Pan et al., 2024). The objective of this Research Topic *Methods in brain-computer interfaces: 2023* is not only to highlight technological advances in BCI, but also to critically assess and share methodological insights that can inform future research design, improve reproducibility, and facilitate practical deployment. This editorial introduces and discusses the key methodologies developed in 2023, emphasizing the methodological advances and their implications for clinical and cognitive applications.

The studies included in this Research Topic each offer unique methodological advances that reflect the diversity and interdisciplinarity of current BCI research. They emphasize methodological contributions—ranging from novel signal processing techniques to integrative paradigms that leverage emerging technologies such as virtual reality, machine learning, and large language models.

Notable advances for improving neural decoding were made, mainly addressing the intra- and inter-subject variability, and the lack of large datasets. Heterogeneous transfer learning was proposed by Feng et al. for improving the generalization of functional near-infrared spectroscopy (fNIRS) classification of motor imagery in stroke patients. To this aim, the authors introduced a cross-subject heterogeneous transfer learning model, based on convolutional neural networks, which leveraged EEG data from healthy individuals as a source domain to improve offline fNIRS decoding in stroke patients. Additionally, reinforcement learning was employed by Fidêncio et al. to dynamically adapt the BCI system to the EEG variability due to inherent non-stationarities, arising from changes in mental state or device characteristics (e.g., electrode placement and impedance). The hybridization of reinforcement learning and BCIs opens exciting avenues for enhancing BCI adaptability, particularly in contexts where a minimal calibration is desirable (avoiding re-calibration due to non-stationarities in long-term BCI use).

Non-invasive decoding of imagined speech from EEG is a frontier application of BCIs, with significant implications for patients suffering from severe motor impairments (e.g., locked-in patients), as it may enable the design of more naturalistic BCIs. Carvalho et al. explored the application of low resource-intensive algorithms for speech decoding based on delay differential analysis, achieving state-of-the-art performance. Non-linear algorithms for speech decoding are widely based on deep learning methods, which are computationally slower, and less robust to noise. Importantly, fast and robust decoders, capable of providing naturalistic EEG decoding, could be valuable for real-world BCI applications.

Finally, Yao et al. introduced a conceptual framework that combines large language models (LLMs), EEG-based BCIs, and virtual reality for improving the diagnosis and the treatment of mild cognitive impairment, seeking to offer patients new avenues for remote diagnostics, treatment, and follow-up care. While the integration of these technologies is in its infancy, this approach theorizes an interesting future direction of BCIs, toward LLM-enhanced BCI frameworks. However, an important challenge of such theorized framework lies in orchestrating its components to operate synchronously, ethically, and securely.

Collectively, these contributions underscore the need for standardized methodologies and comprehensive evaluations of existing techniques to bridge the gap between experimental success and practical implementation. We hope this Research Topic of articles inspires ongoing methodological exploration and interdisciplinary collaboration, fostering the development of more efficient and effective BCI solutions.

## Conclusion

The methodological advances of BCI research in 2023 reflect a field that is aiming to bridge the gap between theoretical innovations and practical utility. From BCI frameworks employing reinforcement learning, heterogeneous transfer learning and LLMs to speech decoding, the year has been marked by a diverse array of breakthroughs. Future research should prioritize a robust benchmarking of the algorithms—also consolidating their validation on larger datasets—and testing online the approaches, to promote the integration of BCIs into real-world applications.
